# A global microbiome survey of vineyard soils highlights the microbial dimension of viticultural *terroirs*

**DOI:** 10.1038/s42003-022-03202-5

**Published:** 2022-03-18

**Authors:** Alex Gobbi, Alberto Acedo, Nabeel Imam, Rui G. Santini, Rüdiger Ortiz-Álvarez, Lea Ellegaard-Jensen, Ignacio Belda, Lars H. Hansen

**Affiliations:** 1 Department of Plant and Environmental Science, University of Copenhagen, Frederiksberg, Denmark; 2 Biome Makers Inc., 95605 West Sacramento, CA USA; 3 Natural History Museum, Centre for GeoGenetics, University of Copenhagen, Copenhagen, Denmark; 4 Department of Environmental Science, Aarhus University, Roskilde, Denmark; 5 Department of Genetics, Physiology and Microbiology, Complutense University of Madrid, 28040 Madrid, Spain

**Keywords:** Microbial ecology, Microbial ecology, Biogeography

## Abstract

The microbial biodiversity found in different vitivinicultural regions is an important determinant of wine *terroir*. It should be studied and preserved, although it may, in the future, be subjected to manipulation by precision agriculture and oenology. Here, we conducted a global survey of vineyards’ soil microbial communities. We analysed soil samples from 200 vineyards on four continents to establish the basis for the development of a vineyard soil microbiome’s map, representing microbial biogeographical patterns on a global scale. This study describes vineyard microbial communities worldwide and establishes links between vineyard locations and microbial biodiversity on different scales: between continents, countries, and between different regions within the same country. Climate data correlates with fungal alpha diversity but not with prokaryotes alpha diversity, while spatial distance, on a global and national scale, is the main variable explaining beta-diversity in fungal and prokaryotes communities. *Proteobacteria*, *Actinobacteria* and *Acidobacteria* phyla, and Archaea genus *Nitrososphaera* dominate prokaryotic communities in soil samples while the overall fungal community is dominated by the genera *Solicoccozyma, Mortierella* and *Alternaria*. Finally, we used microbiome data to develop a predictive model based on random forest analyses to discriminate between microbial patterns and to predict the geographical source of the samples with reasonable precision.

## Abstract (web summary)

A global analysis of vineyard soil microbial diversity provides insights into the biogeographical patterns that characterize wine regions worldwide, capturing the microbial dimension of viticultural *terroirs*.

## Introduction

Wine is a multi-billion dollar market of high cultural and economic value^1^. Since the start of wine history^2^, the place of origin is a major factor driving wine purchase decisions, followed by competitive prices and brands. Winemakers rely on the concept of *terroir* to explain the uniqueness of their wine in terms of taste and flavour. *Terroir* was originally used in Burgundy in the 1930s as a marketing tool to differentiate between wines^3^, but it now goes beyond the wine sector and is used to explain the distinctive regional characteristics of other high-value products, especially those where microbial fermentation has taken place, such as cheese, coffee and cocoa^4,5^. Today, the concept of wine *terroir* has spread around the world, and wine-producing countries are trying to regulate it with the legal definition of appellations of origin. For example, 139 American viticultural areas have been recognised in California (USA) alone^6^ and 90 in Spain^7^. Consequently, protecting the integrity of this classification system is of paramount importance to producers, distributors, retailers and consumers^8^. Thus, a major aim is to establish the scientific basis of wine *terroir*, which relies on multiple dimensions such as local edaphic, climatic, human and biotic factors that contribute to modifying the quality and traits of the resulting wines^9^. Among them, the specific microbial biodiversity associated with the vineyard’s location is reported to be a key aspect associated with biogeographical patterns and directly involved in vine growing, grape quality and winemaking^10^. However, it is still not clear whether the concept of wine *terroir* finds a reliable biological signature in vineyard’s microbiota, and we still do not know its significance at different spatial scales: from local to national and continental scale.

In the wine milieu, pioneering studies conducted by^11–13^ based on high-throughput sequencing (HTS), revealed microbial biogeography associations across multiple viticulture areas. These biogeographical patterns have subsequently been confirmed in other places such as Catalonia in Spain^14^ and in Italy’s Cannonau wine region^15^. Studies into the microbial biodiversity of vineyards also provide relevant insights into the impacts of agricultural management and soil quality^16–18^. Furthermore, Bokulich^1^ and Knight^19^ have shed light on the associations between the microbial and the metabolic fingerprint of wine produced in different wine regions. In this regard, Belda et al.^20^ described distinctive and clustered metabolic patterns for wine-related yeasts depending on their geographical origin. Understanding the links between the unicity of a wine’s metabolic profile and all the factors affecting the grapes is valuable to the viticulturist, and supports the ancient concept of *terroir*. Within this complex and multifaceted concept, the microbiome of vineyards has been shown to be a unique and integrative biomarker^10,21^ that affects wine quality both indirectly (by affecting vine health and physiology) and directly as the main reservoir of autochthonous fermentative microbiota. A comprehensive review on this topic is given by Belda and colleagues^22^. The role of the vineyard’s microbiota in nutrient cycling, plant health, and in all stages of the wine production process, highlights the potential applied impact of microbial *terroirs*. This could serve as a biological objective for future biotechnological applications on targeted regional programmes for pathogens treatment or disease resistance promotion^23^ and, contribute to define biomarkers for monitoring and protecting the biological determinants of wine regions. To achieve these goals, more knowledge is needed on the global vineyard microbiome (at the taxonomic and functional level), how it interacts with biotic and abiotic factors and anthropogenic interventions. In this context, soil biodiversity remains one of the most recognised parameters linked to the concept of sustainable agriculture^24–26^. Since wine grapes are one of the most dramatically affected crops in the current global change scenario^17^, understanding patterns in the microbial community composition of vineyard soils worldwide can potentially advance strategies to manage the sustainability of vitiviniculture.

This study applied an HTS amplicon library sequencing approach to conduct a global survey of the topsoil microbial communities of vineyards in 13 countries, including locations of different wine regions and with different weather conditions, in an endeavour to establish the basis for the development of a global vineyard soil microbiome map. Further modelling efforts on different scales, will provide a better understanding of the role of microbes in connecting vineyard *terroir* with wine quality. Based on the evidence described by Bokulich^1^, Burns^12^, Knight^19^ and Zarraonaindia^11^ between 2013 and 2015, we hypothesised that, although there could be a core microbiome shared between different locations, the link between distinctive microbial communities and specific wine regions is a concept that can be extended to a global scale and can be exploited to distinguish and discriminate between different vineyards location worldwide. Therefore, the aim of this study is to describe the microbial communities of vineyards through amplicon sequencing technology and to build a new microbiome statistical tool to exploit these differences based on a random-forest predictive model. The model we built permits tracing the origin of a given soil sample based only on its microbial community composition allowing a better understanding of the biogeographical basis and the microbiome boundaries of vitivinicultural *terroirs*.

## Results

### Diversity patterns and taxonomic composition of the global vineyard soil microbiome

**Fig. 1:**
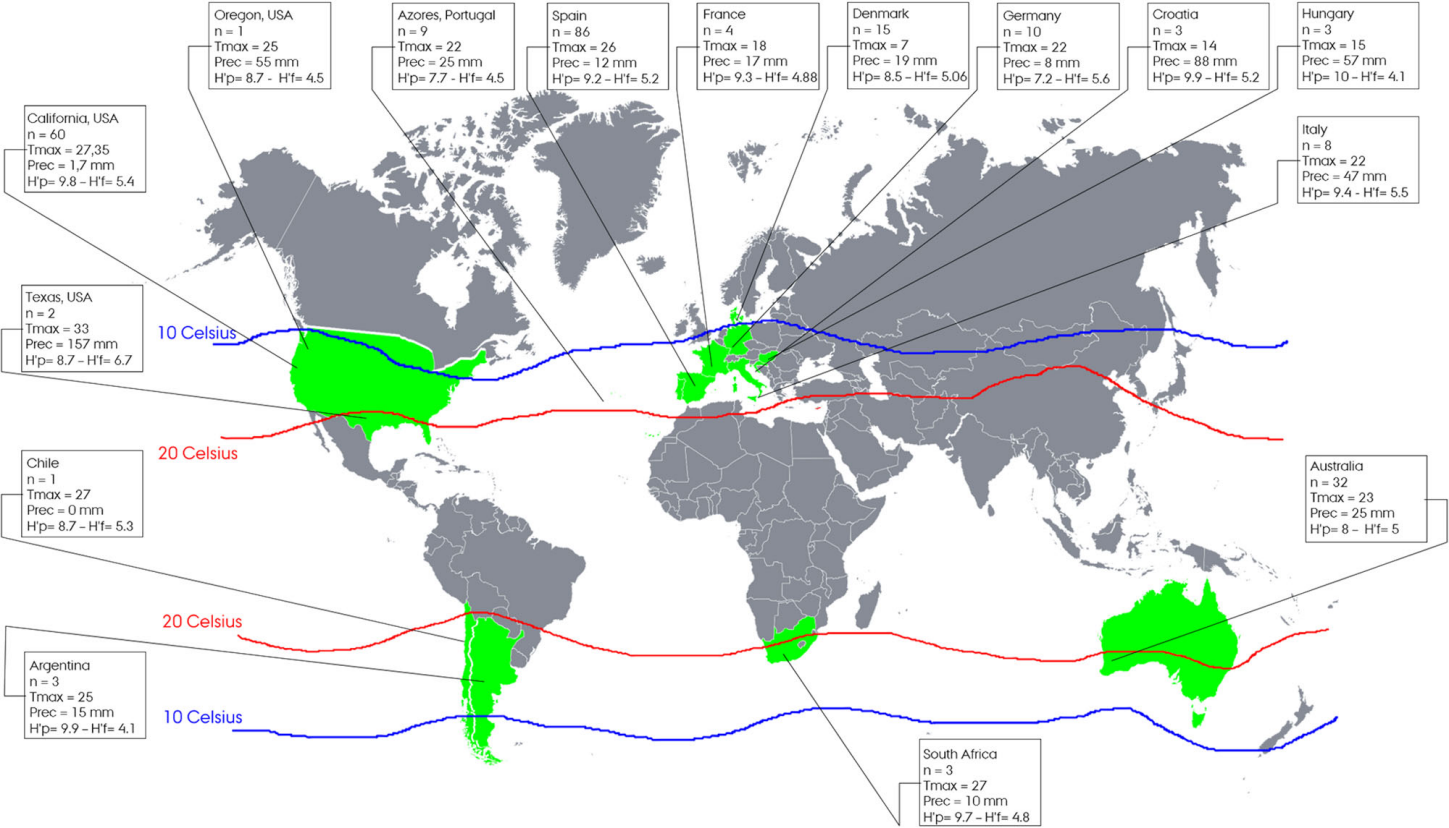
Coverage of the study. The countries highlighted in green are represented in the study’s dataset. The two isothermal lines define the range for optimal conditions for grapevine cultivation (isothermal source: https://www.thirtyfifty.co.uk/spotlight-climate-change.asp). Each panel shows the country, the number of samples (*n*) and information about the average maximum temperature (Tmax) and the level of precipitation (Prec) up to 2 weeks before sample collection. Finally, H′P and H′F define the Shannon Index for the prokaryotic and fungal community respectively.

The Shannon Index for prokaryotes (H′P) and fungi (H′F) are presented in Fig. 1 as the average value for each country. H′P ranged from 7.2 (Germany) to 9.9 (Hungary, Croatia and Argentina), while H′F ranged from 4.1 (Hungary and Argentina) to 6.7 (Texas, USA). An analysis was performed on possible correlations between the Shannon Index in all the different samples and selected meteorological and climatic parameters such as the average maximum temperature and precipitation data prior to sample collection, and long-term climatic data such as the average temperature and rainfalls over the same period. The correlation analyses displayed a significant positive result when fungal H′F was analysed in light of short and long-term average temperature (*r* = 0.38 and *r* = 0.4 respectively) and with short-term rainfall (*r* = 0.52); contrary, no strong correlations (*r* > |0.3 | ) were detected between prokaryotic alpha diversity (H′P) and any short-/long-term climate data. Interestingly, while the correlation between H′F and, short- and long-term average temperature were consistent, only short-term rainfall correlates with fungal H′F alpha diversity. It is important to notice also that the correlation between the short-term average rainfalls, measured close to the sampling, and the long-term average rainfalls within the same period correlate with an *r* = 0.38. This could explain also the different trends that we see between H′F and short vs long-term rainfalls conditions (Supplementary Data 2) and can be due to sporadic local events in that particular vintage or to the different location of the meteorological station providing the data.

Based on their taxonomical annotations, among the 45 prokaryotic phyla identified in these samples, 12 showed relative abundances above 1% in at least one of the 13 countries. *Proteobacteria* occurred with the highest relative abundances for all 13 countries, with values varying between 18.8% (Portugal) and 32.5% (Argentina). *Actinobacteria* were the second most abundant bacterial phylum in most of the countries except Croatia, Germany and Italy where *Acidobacteria* replaced *Actinobacteria* as the second most abundant bacterial phylum, while elsewhere it was the third most represented phylum. Additionally, *Planctomycetes* (Spain and USA)*, Bacteroidetes* (France, Hungary and Croatia) and *Chloroflexi* (Portugal) showed a relative abundance above 10%, and together with *Verrucomicrobia, Firmicutes* and *Gemmatimonadetes* consistently showed a considerable relative abundance, with values above 5%. The archaea phylum *Crenarchaeota* (dominant genus *Nitrososphaera*) showed a large relative abundance variation in the 13 countries, with the main values detected in Portugal (10.9%), Chile (12.5%) and Germany (29.5%), while the values in the other countries ranged between 1.6 and 8.2%. These results are summarised in Fig. S1a.

In the evaluation of fungal taxa detected with relative abundances above 1%, *Solicoccozyma (sin. Cryptococcus)*, when present, was the dominant genus in eight of the 13 countries. The relative abundance of *Solicoccozyma* in Argentina, Chile, South Africa, Italy and Croatia ranged from 13.4 to 39.3%. In the countries where *Solicoccozyma* was not dominant (Australia, Denmark, Germany and Portugal), *Fusarium* and *Cladosporium* were the most dominant. In Portugal and South Africa, *Fusarium* had a relative abundance of up to 10% of the total fungal communities. Denmark almost exclusively displays a high abundance of *Acremonium*. Other abundant fungal taxa can be seen in Fig. S1b.

**Fig. 2:**
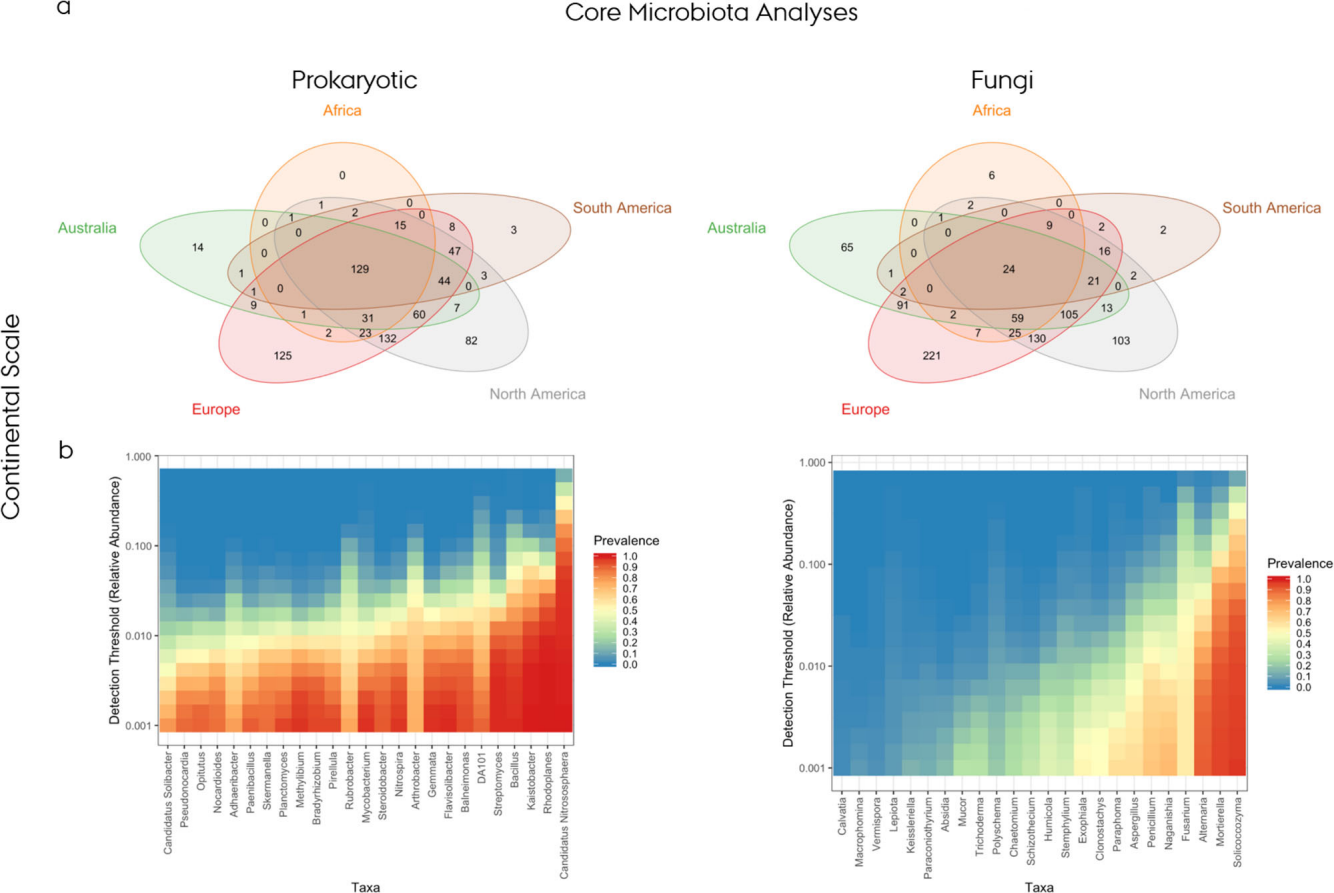
Evaluation of core microbiota. **a** Co-occurrence of different genera constituting the core microbiome at continent-level for the prokaryotic (left) and fungal (right) community visualised with Venn diagrams; **b** Heatmaps for the top 25 genera shared between continents for the prokaryotic (left) and fungal communities (right), including information on their relative abundance and prevalence.

The results outlined in the following section identified the common core microbiota across the vineyard soils and the fractions unique to different areas and are summarised in Fig. 2. The evaluation of the core microbiota, which were consistent across the vineyards regardless of their geographical distance, produced a list of ubiquitous genera (129 prokaryotic and 24 fungal) present in all the countries and in at least 80% of all the samples from each country (Fig. 2a and Supplementary Data 6). From this list, the global prevalence was explored, at different relative abundance levels, of the most widespread distributed genera among the prokaryotic and fungal populations (Fig. 2b). Among the dominant prokaryotic genera were *Nitrososphaera* (Archaea), *Rhodoplanes, Kaistobacter, Bacillus* and *Streptomyces* due to their prevalence at relative abundance values higher than 1%; all these taxa appeared in other papers as normally retrieved in soil and involved in nitrogen cycling, carbon fixation and organic matter degradation^27–32^. Some of them were specifically associated with different types of management (conventional or organic) and future studies confirming their implications could become a predictive signature of the vineyard management system used^32^. Similarly, the main dominant representatives of the core fungal communities were *Solicoccozyma, Morteriella* and *Alternaria* which are mainly involved in degrading organic matter^33–36^. Figure 2b suggests that there was a more diverse and balanced core within the dominant bacteria genera than within the fungal core microbiota, where just three genera seemed to dominate the populations in a substantial proportion of the samples.

### Spatial distance determines the similarity of microbial communities in vineyard soils at different scales

**Fig. 3:**
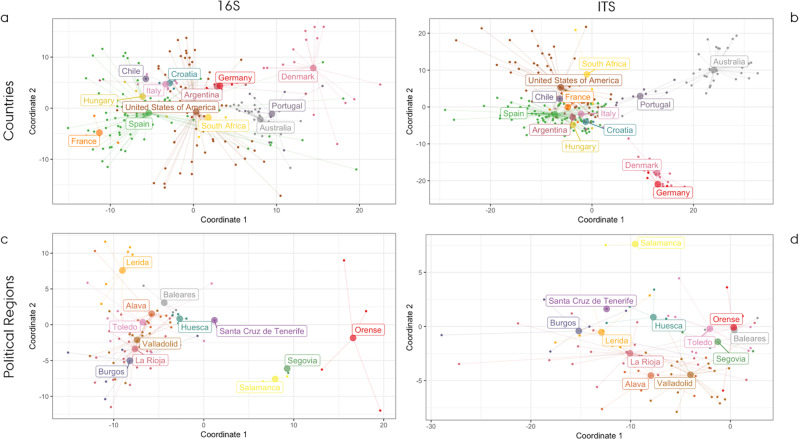
PCoA plots of beta diversity based on CLR. Fig. 3a, b represent the national-scale samples ordination for prokaryotes (**a**) and fungal communities (**b**); Fig. 3c, d represent the national-scale samples ordination for prokaryotes (**c**) and fungal communities (**d**) on a subset of samples from Spain.

Even assuming a certain bias due to batch effect as described in section 2.6, the effect of spatial distance in the composition and structure of microbial communities was visible (Fig. 3) and statistically significant (*p* value < 0.001). Based on the current visualisation, there was a consistent clustering for the individual countries represented in the dataset in both the prokaryotic (Fig. 3a) and fungal communities (Fig. 3b). On a national scale, the most represented country was Spain (*n* = 86) and it was analysed for regional differences. The cluster distribution once again highlighted the different regions sampled (Fig. 3c, d).

The effect of spatial distance on different scales were evaluated using PERMANOVA. The results were consistent with the initial hypothesis confirming an impact of spatial distance on microbial communities in vineyard soils. This was more evident when reducing the scale from a global comparison between continents (prokaryotic: R2 = 0.08, *p* = 0.001; fungal: R2 = 0.09, *p* = 0.001), countries (prokaryotic: R2 = 0.16, *p* = 0.001; fungal: R2 = 0.21, *p* = 0.001), and political regions within a country (prokaryotic: R2 = 0.27, *p* = 0.001; fungal: R2 = 0.25, *p* = 0.001) (Supplementary Data 7).

The impact of geographical distance at different scales (continent, country and region) was significant in all instances except between a few neighbouring underrepresented countries or regions (i.e. Italy and Croatia for 16S or Germany and Denmark for ITS); in order to improve the resolution of the analyses, meteorological data prior to harvest were included as an additional constraint. This effect could be seen when looking at the variance partitioning RDA analyses performed (displayed in Fig. S2), where the use of an additional constraint helped resolve closely related countries. However, as observed in the abovementioned PERMANOVA analysis, it is important to highlight that the variance explained by spatial distance was higher than the variance explained by meteorological data (average maximum and minimum temperatures and precipitation) in the composition of microbial communities, regardless of the scale (Supplementary Data 7). In this context, we assumed that considering additional constraints (e.g. soil physicochemical properties and long-term climate effect) would increase the resolving power of this approach.

For a practical demonstration of the effect of geographical distance in determining the structure of microbial communities in vineyard soils as a key consideration when defining wine *terroirs*, a predictive model was developed based on random forest analyses. The objective was to trace the origin of a certain soil sample based solely on its microbiome composition. The fitted models for each level (country or continent), sequencing type (ITS or 16S) and inclusion of weather variables had test set accuracies of between 80.0% and 93.3%. Test set accuracies for the final models are presented in Supplementary Table 2. The continent-level models were more accurate compared with country-level models, regardless of the inclusion of weather conditions in the analysis. However, quite notably, the inclusion of weather variables did not seem to have a large effect on test set accuracy, when used in conjunction with sequencing data.

**Fig. 4:**
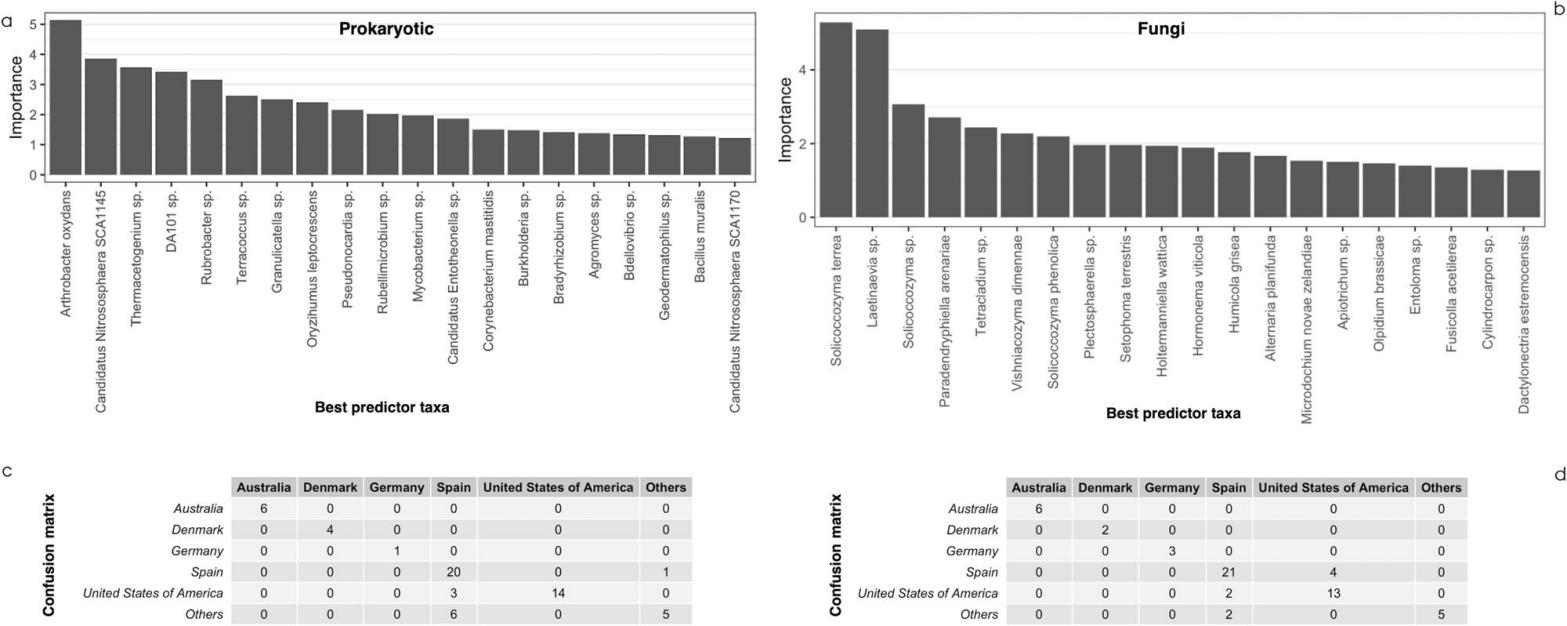
Random forest results at national scale including only microbial data. The top charts show the 20 best predictors for prokaryotic (**a**) and fungal (**b**) communities; the bottom charts show the confusion matrixes for the prokaryotic (**c**) and fungal communities (**d**).

Figure 4 shows the confusion matrix reporting the predictions from the trained models. As mentioned above, the reasonable accuracy of the fitted models resulted in a high rate of coincidence between the actual and predicted origins of the samples, with a similar performance of prokaryotic and fungal-based models. Figure 4b shows the taxonomical identity of the 20 best predictor phylotypes. As shown in Fig. S3, weather data did not have a large effect on test set accuracy, and microbial variables remained the main predictors in these mixed models.

## Discussion

This work provides new insights into the microbial community of vineyard soils worldwide such as the correlation between microbial diversity and environmental factors, the impact of spatial distance on a multi-scale perspective, the identification of a global core-microbiome (Supplementary Data 6) and the development of a prediction model by analysing amplicon data for bacterial 16S rRNA gene and fungal ITS region sequencing from different wine-producing countries. The PCoA plots confirmed the link between spatial distance and microbial community in vineyards on global and regional scales. There has been few evidences of this on a regional scale in California^37,38^, New Zealand^19^, Chile^39^, Italy^15,40^ and Australia^41^. The present study extends the concept of biogeographical correlations in the vineyard, within the framework of microbial *terroir*^21^, to a global scenario. Spatial distance has a strong effect on shaping the microbial community of vineyard soils from different countries, with few clusters overlapping possibly due to the relatively high variance between the microbial community retrieved from different vineyards in the same country. This led to the suggestion that there is a hierarchy within spatial distances, with the general trend being the further the distance, the more diverse the community. The present study confirmed the regional-scale clustering when looking at the samples from Spain (Fig. 2c, d). This was the case for bacteria and fungi on an international scale but was even more noteworthy when looking at the distribution within the same country. This suggests that the use of microbial information is a sensitive parameter for discriminating between more closely located communities when confounding factors are reduced.

When taking into account the correlations between Shannon Index, calculated at the national level, and the short and long-term weather conditions at the sampling location around the harvest, results have shown some trends consistent with the existing literature. A positive correlation between temperature and fungal diversity is expected due to the direct and indirect effects represented by a lower pH and a positive effect on microbial-rate metabolism^42^. In a similar way, the positive correlation between rainfalls and fungal diversity could be explained by changing resource availability and influencing microbial metabolic activity^43^. Furthermore, Větrovský et al. in 2019^44^ showed that, among different environmental factors, the climate is a strong driver of fungal diversity, which tends to increase with latitude (while other studies suggested the opposite i.e. Arnold et al. in 2010^45^) but have a wider range of distribution in the temperate area included in this study. Specifically, the strongest drivers they identified were the mean temperature of the driest quarter (generally recorded in summer which corresponds to the timing of our sampling) and precipitation seasonality which was also recorded in our study. The positive correlation between fungal diversity and rainfall is also supported by Tedersoo et al.^46^ that in 2014 described the relations between a global fungal distribution and environmental factors in different biomes.

At a taxonomic level, *Proteobacteria* and *Acidobacteria* have previously been identified as the most abundant bacterial phyla within soils^47^, including topsoil studies carried out in vineyards^11,37,41^. In agreement with such observations, *Proteobacteria* was the most abundant phylum in all 13 countries evaluated. The widespread occurrence of *Crenarchaeota* members in these samples is in agreement with the detection of this archaeal phylum in a range of environments around the world, such as agricultural fields, sandy soil, forest soil, contaminated soil and the rhizosphere^48,49^. However, while the relative abundance fraction of this phylum is typically reported as representing up to 5% of the total prokaryotic community^50,51^, it was found in 10 of the 13 countries in values exceeding 5%, with an emphasis on samples from Chile (12.6%) and Germany (29.4%). The high abundance of *Crenarchaeota* seen here in samples from Germany (Fig. S1) has also been identified by^52^, but in their case in samples of acidic forest soil. In contrast to the present observation, studies of soil samples from Chilean vineyards have previously reported relative abundances of *Crenarchaeota* below 0.1%^39^. For *Crenarchaeota*, the ammonia-oxidising archaea (AOA) *Nitrososphaera* was the main genus detected in all the countries surveyed in this study. This genus has been observed by^53^ to significantly respond to agricultural management practices. In the present study, this taxon was also one of the best predictors from the random forest model.

Of the fungi, *Solicoccozyma* (mostly known as *Cryptococcus*) was the dominant genus in eight out of the 13 countries and the best predictor in the random forest model considering fungal community alone. It belongs to the group of oxidative basidiomycetous yeasts and has been found to be associated with the phyllosphere, grapes and soil^54^ involved in wood-decomposition^55^. *Cryptococcus*, *Saccharomyces* (Spain and USA) and *Candida*, *Hanseniaspora* and *Pichia* (these last three genera were not identified in the samples in the present study) provide most of the diversity of the frequently isolated yeast species related to grape or isolated from fermented grape juice^56^. Studies have shown that *Cryptococcus* are not severely affected by fungicides, which may explain their higher abundance in vineyards^57,58^. Some of the other main fungal taxa identified in the present samples also contained documented plant pathogens at a lower resolution level. *Fusarium*, detected among the dominant taxa in Australia, Denmark, Germany, Portugal and South Africa, is recognised as containing many plants pathogenic and fruit spoilage species^59^. However, the detection of these potential phytopathogens does not directly indicate plant infection because the success of microorganisms to affect plant health relies on whole soil and rhizosphere microbial interactions^60^ and strain-specific virulence factors that cannot be retrieved with amplicon sequencing. In Australia, the presence of soil-borne *Fusarium* has been also detected among the dominant taxa on leaves^61^.

Based on the random forest results, both 16S and ITS abundances showed good predictive power for the geographical region. Overall, accuracies were 83 and 86% on a national scale for the prokaryotic and fungal communities respectively (Supplementary Table 2). These results included the list of the best predictor taxa, some of which are also known to play an ecological role in vineyards, such as *Nitrososphaera* and *Cryptococcus*, which also appeared as the most dominant taxa within the prokaryotic and fungal core communities (Fig. 3b). The few occasions on which the confusion matrix gave a misprediction could be attributed to several factors; one was due to the approximation applied to overcome the different coverage of the US states represented in the dataset. In fact, although all the samples from the United States of America were considered to be from a single country, they were collected in three different states (California, Oregon and Texas) which increased the dispersion and group variance leading to a model mistake. Finally, apart from all the regional signatures described above, it should be also highlighted that a core microbiome could be identified across vineyard soils, as previously described in other global surveys^62,63^.

To conclude, this study has provided new insights into microbial biogeographical correlations, quantifying them on different scales for the first time to our knowledge. This concept was extended to a global scale, showing a hierarchical effect that is valuable on continental, national and regional scales. The level of the resolution reached here, together with some other evidence reported at a local (inter-block and intra-block) scale^64^, suggests that the microbiome should be considered as an important variable in identifying agricultural sites for the definition of homogeneous functional zones such as basic *terroir* units. These should represent the smallest area for which it is possible to objectively describe the effect of the environment on plant physiology and agricultural production, and which could be differentially managed. Since the microbial *terroir* appears to be dependent on several different factors, from geography to climate, soil characteristics and vineyard management, there is a thread that links them all and this must be sought in the hidden dynamics of their microbial communities. Thus, the use of microbial information, as a way of discriminating between vineyards in different countries, provides the first applicative use of this technology and tools for improving the accuracy and representativeness of the microbial map by adding new samples in the future. The random forest model developed ultimately confirmed this study’s initial hypothesis that spatial distance determines the microbiota to such an extent that it can be used to predict the origin of a vineyard’s soil. This is therefore another argument supporting the definition of appellations of origins, both in legal and marketing terms. Finally, these results should encourage further explorations of the significance and limits of the microbial aspect of agricultural *terroirs*, since they provide a baseline for guiding future studies in the field. In this sense, we should make explicit the main limitations of our work which, indeed, still represent the knowledge gaps that should be addressed in future studies in agreement with the recommendations of the International Organization of Vine and Wine (OIV) on its recent resolution OIV-VITI 655-2021^65^: (i) the importance of soil physical-chemical properties, farming practices, and long-term climate data in shaping the vineyard microbiome; (ii) the role of plant–soil–microbe interactions in filtering the microbiota which finally occupies the rhizosphere; (iii) the identification of keystone taxa and the role of facilitators and competitors in promoting a resistant and resilient soil microbiome in vineyards.

## Methods

### Materials

This study involved a microbial amplicon-based survey created with previously unpublished data combined from two different datasets. The data in this study originated partly from the MICROWINE project but also included private data from Biome Makers Inc. obtained with BeCrop^®^ Technology. This project involved a total of 252 topsoil samples from 200 vineyards collected close to harvest. These samples were thus collected by different people around the globe between 2015 and 2018. Although there were some differences in the sampling scheme, storage conditions and sample processing, a general description of this protocol follows: all samples were bulk-topsoil, collected in sterile tubes at a depth of between 0 and 10 cm. The MICROWINE samples consisted of five samples collected and DNA sequenced for each field. The Biome Makers’ samples consisted of pooled bulk-topsoil from three random spots in each field, and DNA was then extracted and sequenced for this composite sample. The shipment conditions of the soil samples were either −20 °C or non-refrigerated, followed by −20 or −80 °C as the storage temperature in the laboratories until DNA extraction. DNA extractions were performed using bead beating-based DNA extraction kits such as the Dneasy Powerlyzer Powersoil Kit (Qiagen) for the BeCrop^®^ platform (patent publication number: WO2017096385, Biome Makers) and the FAST-DNA Spin Kit for Soil (MP-Biochemical) for the MICROWINE project. A complete overview of all the samples used in this study and their relative origin is given in Supplementary Data 1. The use of bead beating-based kits, such as those included in this study, ensured that the results produced were comparable and also allowed the recovery of the highest biodiversity within soil samples^66^.

### Dataset

The sequencing dataset obtained, made of 504 samples equally divided between 16S and ITS, was representative of 252 soil samples collected in 200 locations across 13 different countries on four continents, with the intention of covering most of the dominant areas for grapevine cultivation (Fig. 1). These 13 countries represent more than 83% of total wine production worldwide^27^. The collection time, close to the harvest period, allowed the dataset to be built in a way that was strongly dependent on the geography and included vintage-related parameters such as average maximum and minimum temperatures and precipitation measured for the two weeks prior to sample collection. Analyses of the microbial composition on a country-level scale were performed using the samples from Spain, the country with the highest number of samples (*n* = 86) from 12 wine regions. A total of 84770 non-redundant amplicon sequence variants (ASVs) for 16S and 33254 for ITS were obtained.

Weather information were retrieved from World Weather Archive (https://www.worldweatheronline.com/) with the closest reference point chosen based on the GIS coordinates identifying the samples and reported in Supplementary Data 1. Long-term climatic data are retrieved from World Climate Archive (http://www.worldclimate.com/) with the closest reference point chosen based on the GIS coordinates identifying the samples and reported in Supplementary Data 1.

The data are publicly available via European Nucleotide Archive (ENA) under the following study accession number: PRJEB40350.

### Library preparation

The same variable regions were investigated in both datasets (MICROWINE and Biome-Makers) for 16S and ITS with a few differences in the primer sequences. A complete description of the primers is given in Supplementary Table 1. All PCR reactions were prepared using UV-sterilised equipment and negative controls were run alongside the samples. Furthermore, PCR conditions, such as the number of cycles, annealing temperature, thermocycler and Master Mix composition, changed between samples from different projects. The libraries for both MICROWINE datasets (16S and ITS) were prepared using a two-step PCR, as described by^67^ and^28^ Biome Makers samples were obtained amplifying the 16S rRNA V4 region, while the ITS were obtained by amplifying the ITS1 region using BeCrop^®^ custom primers (patent WO2017096385). All libraries were prepared following the two-step PCR Illumina protocol and these were subsequently sequenced on an Illumina MiSeq instrument (Illumina, San Diego, CA, USA) using 2 × 251 paired-end reads for the MICROWINE samples and 2 × 301 paired-end reads for the Biome Makers samples.

### Bioinformatics

All the data produced and collected were subsequently analysed using QIIME2 v2019.7^68^, as described in ref. ^28^. Reads from the 16S rRNA gene produced following the MICROWINE and Biome Makers protocol were collected and processed using DADA2 single-read analyses^69^. The phylogenetic tree was calculated based on the insertion fragment plugin to reduce the batch effect from using two different primer sets^70^. Taxonomy was assigned using a Naive Bayes classifier^71^ trained with Greengenes v13_8^72^. ITS sequences were analysed using a DADA2 single read without merging. Denoised reads were used to build a phylogenetic tree using MAFFT^73^ and subsequently taxonomy was assigned using a Naive Bayes classifier trained with the UNITE database (full alignment)^74^. The frequency tables for 16S and ITS were rarefied to 10,000 high-quality reads per table and used for all subsequent analyses of diversity and composition.

### Statistics and reproducibility

Alpha diversity was calculated using the Shannon Index and tested with a Kruskal–Wallis test with adjustment for multiple testing. Alpha diversity correlation with the short-term weather parameters and long-term climatic data was performed using Pearson’s correlation coefficient on Microsoft Excel. An arbitrary threshold of *r* = 0.3 was considered relevant if supported by a *p* value lower than 0.05. These results are reported in Supplementary Data 2. Beta diversity was calculated based on unweighted UNIFRAC^75^. Kruskal’s non-metric scaling was used to perform a principal coordinate analysis based on UNIFRAC distances between samples. The results were plotted, labelling samples by country and continent. These groupings were tested with PERMANOVA. All the information about the explained variance for the different groups of variables, with PERMANOVA scores, is reported in Supplementary Data 3. To assess the global core microbiome, taxa were preselected if they were detected in each continent. This was visualised as a Venn diagram using the Venn Diagram R package^76^. These ‘core’ taxa were evaluated on a continuum of abundances and prevalences and plotted as a heatmap as per ref. ^77^. For subsequent analyses, taxa that had fewer than 20 non-zero abundances in the whole dataset were aggregated into an ‘others’ variable. A zero replacement was also performed using Bayesian inference with a Dirichlet prior and multiplicative adjustment to maintain other proportions^78^.

A redundancy analysis (RDA) was performed using a centred log-ratio (CLR) of 16S and ITS abundances, constrained by the country or continent from where the samples came. Additionally, weather conditions (specifically average minimum and maximum temperatures and precipitation) at sampling were used as conditioning variables for RDA. The RDA analysis was conducted using the vegan R package (Oksanen et al.^79^).

The predictive potential of the microbiome for the geographical region was assessed using random forests. Models were fitted using CLR-transformed 16S and ITS abundances as predictors and country or continent as an outcome. Additional random forests were fitted using weather variables (as mentioned above) in conjunction with abundances as predictors. Subsequently, 75% of the dataset was randomly selected and used for repeated cross-validation, using a grid of values for hyper-parameters (Supplementary Data 4). Models were selected for the highest accuracy in the test set (remaining 25% of the dataset). Country and continent-level confusion matrices were generated for the test set (Supplementary Data 5). Feature ranking was performed using the Gini index. Random forests were fitted using the ranger R package^80^. All the analyses were performed in the R programming environment (R Core Team^81^) and Qiime2 v2019.7^68^. The limitation of this approach is that convenience sampling may affect the generalisability of this model. However, this does not affect the conclusion of this study as it aims to train a model to distinguish between source locations given microbiome data rather than predicting new samples' microbial composition. Additional constraints such as soil physicochemical properties and vineyard management system have not been included in this project and this limits the conclusions we could draw to those reported in this study.

When combining datasets from different studies, the impact of what is known as batch effect bias needs to be addressed. The database in this study was composed of samples from several countries (see the detailed list of samples included in this study in Supplementary Data 1), and although they were produced in a similar way, confounding factors could be expected in the global distribution. The batch effect is a very common bias, especially in meta-analytical studies^82^, that can influence the results and subsequently the conclusions. This risk is even higher when the same type of sample is compared, while the effect is lower when different types of samples are analysed together, as reported by several meta-analyses^82–84^. With regard to the 16S rRNA gene analyses, an algorithm called SEPP^70^ was chosen that has been shown to dramatically reduce the bias due to the use of different primers or to the amplification of different hypervariable regions. This method is not applicable to ITS analyses, since the lack of a high-quality phylogenetic tree will affect the results more than a traditional pipeline. For ITS, a single-read DADA2 approach with basic filters was used instead that has been shown to represent one of the best approaches for amplicons so far^85^ and has also previously been used^86^.

On a global scale, alpha diversity was measured using the Shannon Index, which is relatively stable against the batch effect from the different primers used. This is because it also accounts for evenness in the ASVs distribution and is not solely based on their presence/absence^87^. Furthermore, once singletons are removed, its entropy coverage adjustment accounts for unobserved taxa caused by an uneven coverage of the countries^88^. Another approach was to compare the co-occurrence pattern within the whole database after taxonomy assignment at different taxonomical ranks^87^. In fact, two ASVs that have a different nucleotide sequence, coming from different regions of the same gene marker, could be assigned to the same taxa, despite some differences that can occur in relative abundance.

### Reporting Summary

Further information on research design is available in the Nature Research Reporting Summary linked to this article.

## Supplementary information

Supplemental material

Description of Additional Supplementary Files

Supplementary Data 1

Supplementary Data 2

Supplementary Data 3

Supplementary Data 4

Supplementary Data 5

Supplementary Data 6

Supplementary Data 7

Reporting Summary

## Data Availability

The sequencing data are available via European Nucleotide Archive (ENA) under the following study accession number: PRJEB40350. The authors declare that the other data supporting the findings of this study are available in the Supplementary Information file and Supplementary Data 1–7.
