# Supplementary material for: A global microbiome survey of vineyard soils highlights the microbial dimension of viticultural terroirs
Source: Commun Biol. 2022 Mar 18;5:241. doi: 10.1038/s42003-022-03202-5 (PMC8933554; doi:10.1038/s42003-022-03202-5)
Supplement: Supplementary file 1 — Supplemental material [file 42003_2022_3202_MOESM1_ESM.pdf]

A global microbiome survey of vineyard soils highlights the microbial dimension of viticultural *terroirs*

Alex Gobbi[1], Alberto Acedo[2], Nabeel Imam[2], Rui .G. Santini[3], Rüdiger Ortiz-Álvarez[2], Lea Ellegaard-Jensen[4], Ignacio Belda[2,5*¥] Lars .H. Hansen[1¥]

[1] Department of Plant and Environmental Science, University of Copenhagen, Denmark
[2] Biome Makers Inc., 95605-West Sacramento, CA, USA
[3] Natural History Museum, Centre for GeoGenetics, University of Copenhagen, Copenhagen, Denmark
[4] Department of Environmental Science, Aarhus University, Roskilde, Denmark
[5] Department of Genetics, Physiology and Microbiology. Complutense University of Madrid, 28040-Madrid, Spain

¥ IB and LHH supervised the project equally

* corresponding author

Supplementary Information

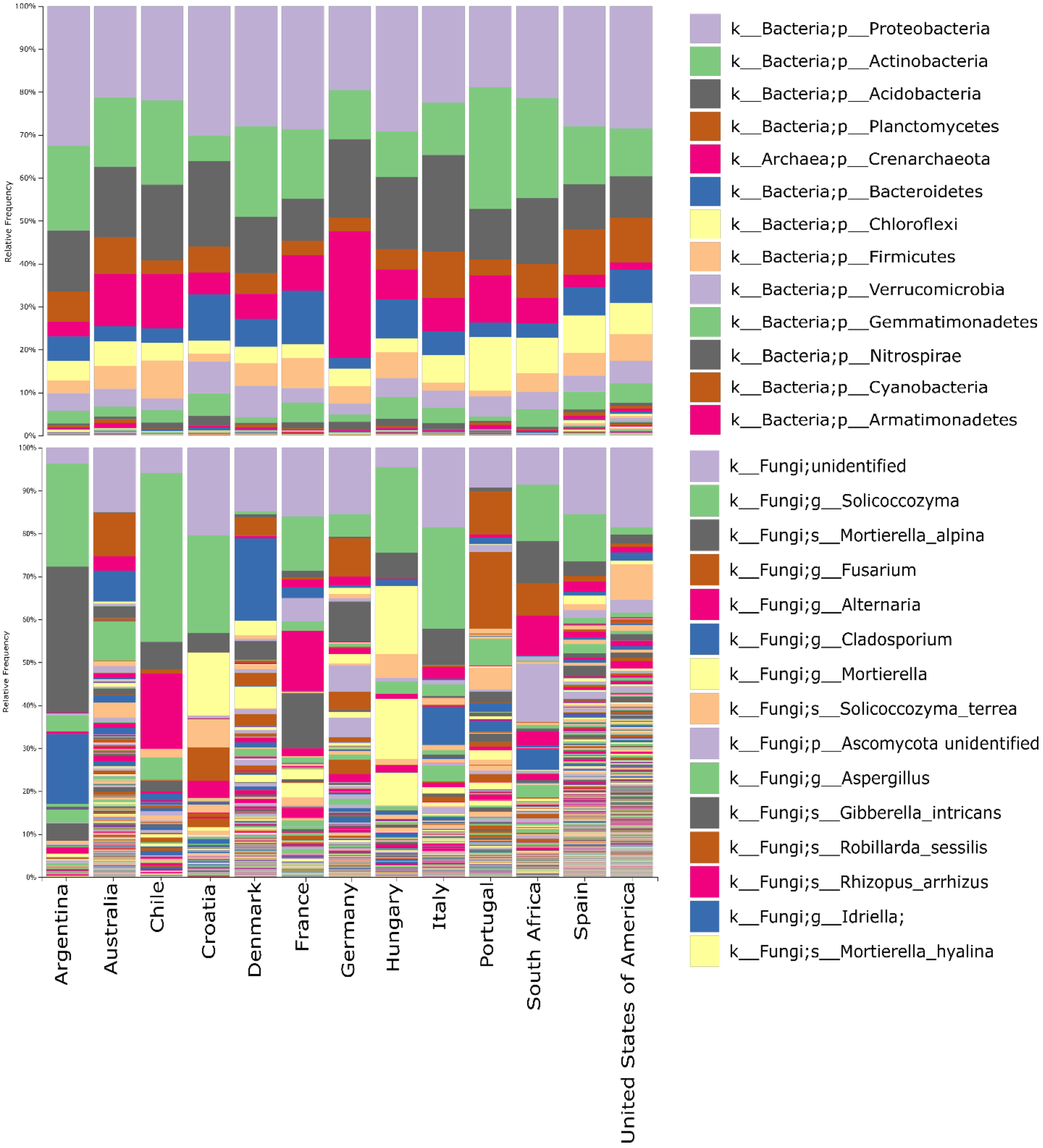

Figure S1. Taxonomical composition at country level: above) a phyla-level taxa bar plot representing the samples' 16S community, grouped by country; the colours identify the 13 most represented phyla. Below) a taxa bar plot representing the fungal community at the highest rank for the 15 most represented taxa in the samples, grouped by country.

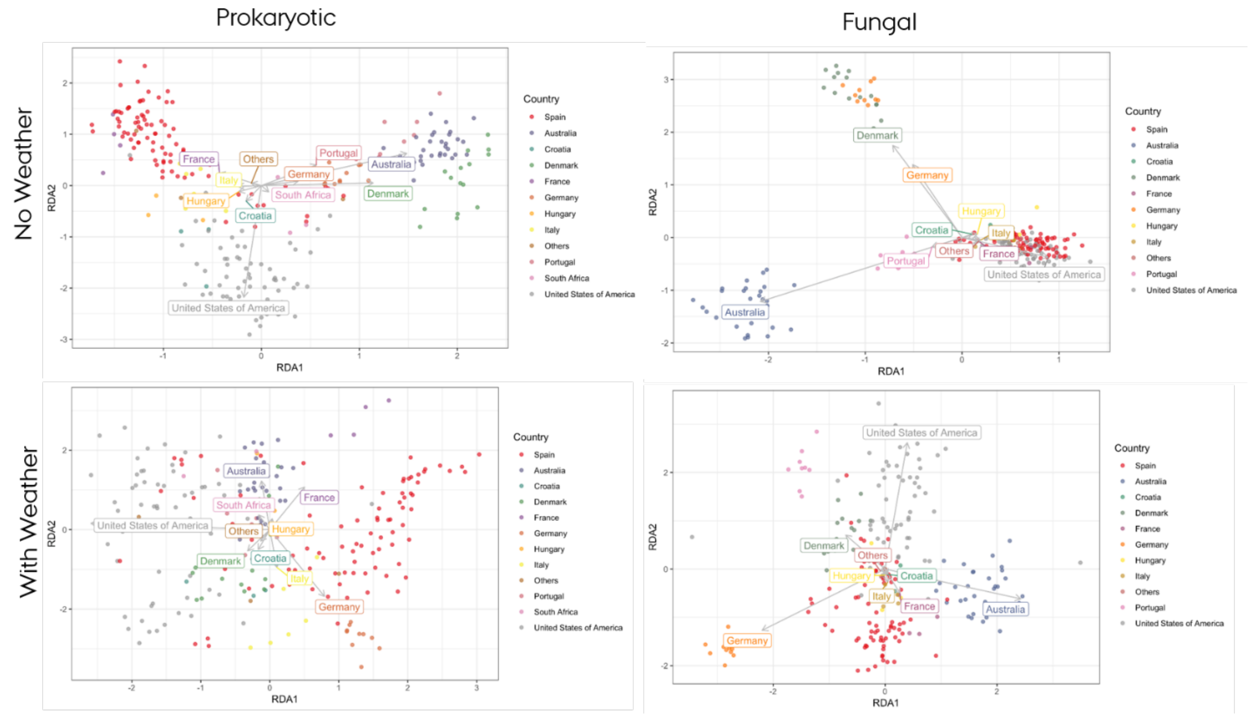

Figure S2. Variance partitioning RDA on the prokaryotic and fungal community with and without weather conditions. Samples are coloured based on the countries they come from.

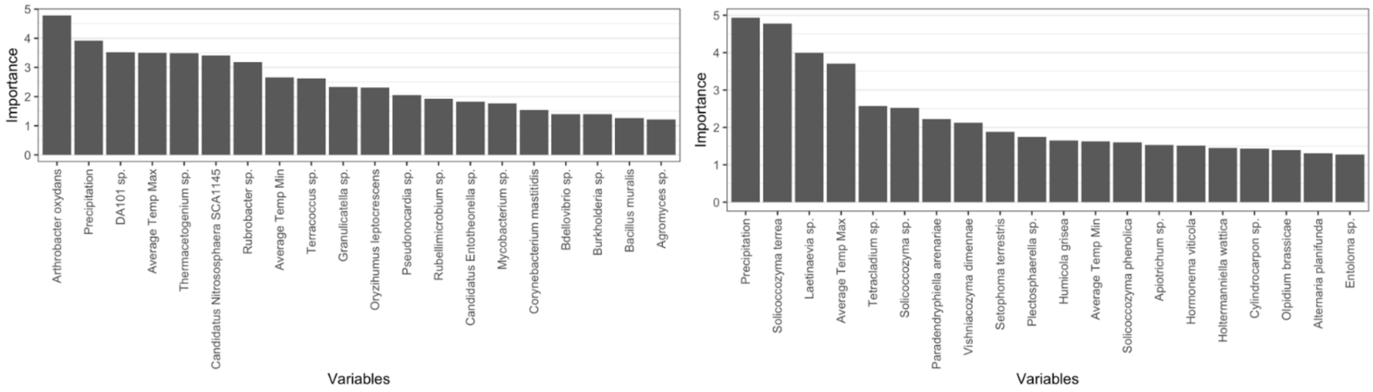

	Australia	Denmark	Germany	Spain	United States of America	Others
Australia	6	0	0	0	0	0
Denmark	0	4	0	0	0	0
Germany	0	0	1	0	0	0
Spain	0	0	0	19	0	2
United States of America	0	0	0	3	14	0
Others	0	0	0	7	0	4

	Australia	Denmark	Germany	Spain	United States of America	Others
Australia	6	0	0	0	0	0
Denmark	0	2	0	0	0	0
Germany	0	0	3	0	0	0
Spain	0	0	0	24	0	1
United States of America	0	0	0	2	12	1
Others	0	0	0	2	0	5

Figure S3. Random forest results at national scale including microbial and meteorological data. The top charts shows the confusion matrixes for the prokaryotic and fungal communities; the bottom charts give the 20 best predictor for prokaryotic (right) and fungal (left) community.

Supplementary Tables 1: Primers used in this study for 16S and ITS

Sequence 5'-3'	Name	Region	Amplicon	Study	Reference
GTGYCAGCMGCCGCGGTAA	515F	V4	16S	MW	(Caporaso et al. 2011)
GGACTACNVGGGTWTCTAAT	806R	V4	16S	MW	(Caporaso et al. 2011)
Available upon request	/	V4	16S	BM	Patent WO2017096385
Available upon request	/	V4	16S	BM	Patent WO2017096385
Available upon request	/	ITS1	ITS	BM	Patent WO2017096385
Available upon request	/	ITS1	ITS	BM	Patent WO2017096385
GAACCWGCGGARGGATCA	ITS1IF2	ITS1	ITS	MW	(Gaylarde et al. 2017)
GCTGCGTTCTTCATCGATGC	ITS2	ITS1	ITS	MW	(Gaylarde et al. 2017)

Supplementary Tables 2: Random Forest Model Accuracy for each Test-Dataset according to the confusion Matrix

Test	Type	Level	Weather Conditions	Test_set_accuracy	Test_Set_Accuracy_%
1	16s	Country	yes	0.8	80
2	16s	Country	no	0.833333333	83.3333333
3	16s	Continent	yes	0.916666667	91.6666667
4	16s	Continent	no	0.933333333	93.3333333
5	its	Country	yes	0.896551724	89.6551724
6	its	Country	no	0.862068966	86.2068966
7	its	Continent	yes	0.913793103	91.3793103
8	its	Continent	no	0.913793103	91.3793103
