# Supplementary material for: A global microbiome survey of vineyard soils highlights the microbial dimension of viticultural terroirs
Source: Commun Biol. 2022 Mar 18;5:241. doi: 10.1038/s42003-022-03202-5 (PMC8933554; doi:10.1038/s42003-022-03202-5)
Supplement: Supplementary file 2 — Description of Additional Supplementary Files [file 42003_2022_3202_MOESM2_ESM.pdf]

Description of Additional Supplementary Files

File name: Supplementary Data 1
Description: sample name deposited on Sequence Read Archive and corresponding environmental data used in this study

File name: Supplementary Data 2
Description: Raw data and correlations between: Long-Term Climate, Weather close to Harvest and Alpha-Diversity

File name: Supplementary Data 3
Description: Explained variance PCA Plots for 16S and ITS at continental, national and regional scale distribution; The variance explained by both axes is measured in the R2 column while the PERMANOVA score is reported in the column Pr(>F).

File name: Supplementary Data 4
Description: List of Best-Predictors for the Random Forest Model that were determined by the model and used to assign the samples to their country based on microbial composition, with and without weather conditions

File name: Supplementary Data 5
Description: Confusion Matrix from Random Forest Model on Test-Dataset; numbers in each cells represent the calls of the model for each iteration

File name: Supplementary Data 6
Description: Complete list of the genera for the prokaryotic and fungal community that constitutes the core-microbiome at Continental-level based on our dataset.

File name: Supplementary Data 7
Description: PERMANOVA Results based on Unweighted Unifrac Distance repeated for each gene-marker, scale and with/without weather conditions
