# Supplementary material for: A global microbiome survey of vineyard soils highlights the microbial dimension of viticultural terroirs
Source: Commun Biol. 2022 Mar 18;5:241. doi: 10.1038/s42003-022-03202-5 (PMC8933554; doi:10.1038/s42003-022-03202-5)
Supplement: Supplementary file 10 — Reporting Summary [file 42003_2022_3202_MOESM10_ESM.pdf]

Corresponding author(s): Ignacio Belda

Last updated by author(s): Jan 17, 2022

Reporting Summary

Nature Research wishes to improve the reproducibility of the work that we publish. This form provides structure for consistency and transparency in reporting. For further information on Nature Research policies, see our Editorial Policies and the Editorial Policy Checklist.

Statistics

For all statistical analyses, confirm that the following items are present in the figure legend, table legend, main text, or Methods section.

n/a	Confirmed	
☐	☒	The exact sample size (*n*) for each experimental group/condition, given as a discrete number and unit of measurement
☐	☒	A statement on whether measurements were taken from distinct samples or whether the same sample was measured repeatedly
☐	☒	The statistical test(s) used AND whether they are one- or two-sided<br>*Only common tests should be described solely by name; describe more complex techniques in the Methods section.*
☐	☒	A description of all covariates tested
☐	☒	A description of any assumptions or corrections, such as tests of normality and adjustment for multiple comparisons
☐	☒	A full description of the statistical parameters including central tendency (e.g. means) or other basic estimates (e.g. regression coefficient) AND variation (e.g. standard deviation) or associated estimates of uncertainty (e.g. confidence intervals)
☐	☒	For null hypothesis testing, the test statistic (e.g. *F*, *t*, *r*) with confidence intervals, effect sizes, degrees of freedom and *P* value noted<br>*Give P values as exact values whenever suitable.*
☒	☐	For Bayesian analysis, information on the choice of priors and Markov chain Monte Carlo settings
☒	☐	For hierarchical and complex designs, identification of the appropriate level for tests and full reporting of outcomes
☒	☐	Estimates of effect sizes (e.g. Cohen's *d*, Pearson's *r*), indicating how they were calculated

Our web collection on statistics for biologists contains articles on many of the points above.

Software and code

Policy information about availability of computer code

Data collection	No original or unpublished software was used for data-collection. Weather information was retrieved from World Weather Archive (https://www.worldweatheronline.com/), and long-term climatic data was retrieved from World Climate Archive (http://www.worldclimate.com/) with the closest reference point chosen based on the GIS coordinates identifying the samples.
Data analysis	All the data collected in this study were analyzed using Qiime2 v2019.7 and R-Core-Team-2019

For manuscripts utilizing custom algorithms or software that are central to the research but not yet described in published literature, software must be made available to editors and reviewers. We strongly encourage code deposition in a community repository (e.g. GitHub). See the Nature Research guidelines for submitting code & software for further information.

Data

Policy information about availability of data

All manuscripts must include a data availability statement. This statement should provide the following information, where applicable:

- Accession codes, unique identifiers, or web links for publicly available datasets
- A list of figures that have associated raw data
- A description of any restrictions on data availability

The data are publicly available via European Nucleotide Archive (ENA) under the following study accession number: PRJEB40350

Field-specific reporting

Please select the one below that is the best fit for your research. If you are not sure, read the appropriate sections before making your selection.

☐ Life sciences ☐ Behavioural & social sciences ☒ Ecological, evolutionary & environmental sciences

For a reference copy of the document with all sections, see nature.com/documents/nr-reporting-summary-flat.pdf

Ecological, evolutionary & environmental sciences study design

All studies must disclose on these points even when the disclosure is negative.

Study description	We sampled 252 soil-samples from 200 vineyards. We built 504 amplicon libraries equally distributed between 16S and ITS and subsequently analyzed them to retrieve information about the microbial community harboured in the vineyard's soil. We developed a model based on random forest to trace-back a soil sample to its country of origin only based on its microbial composition.
Research sample	The research samples used in this research were soil samples taken form the top-surface 0-5cm in vineyards from 13 different countries worldwide. The choice of soil is due to the fact that it has been shown to be the main reservoir of microbes colonizing grapevine plants. The choice of the depth is due to the fact that top-soil harbor the highest microbial biomass and diversity. Finally the 13 countries represented in this study account for the biggest wine-producers worldwide
Sampling strategy	Existing studies in literature describe analogous way of collecting samples to represent microbial population within a field. In this studies two different dataset were produced. In one dataset (BM) three samples were taken randomly within each field and subsequently pooled together before the analyses. In the other dataset (MW) 5 samples were taken in the field following a diagonal-scheme pattern. Samples for both dataset are taken from top-soil (0-5 cm) and collected within two weeks from the grape-harvest to have consistent climatic conditions between countries
Data collection	Samples were taken and processed by AG and AA during three years from 2015 to 2018. The whole data-analyses started in 2018
Timing and spatial scale	The samples collection was planned and performed accordingly to harvest-time in the 13 different countries across the different years of sampling. The countries were chosen due to their importance in the wine-production
Data exclusions	spurious samples that was lacking either the bacterial or the fungal representation were excluded from the analyses. This was done in order to allow future interaction-studies using the same dataset. Also, samples that contained less than 10000 reads for each genetic marker were excluded from the analyses.
Reproducibility	All the attempts to repeat the experiment were successful. DNA extraction methods, library preparation protocols and subsequent sequencing are successfully applied on all the samples included and excluded from this research
Randomization	The samples were collected by different people, processed during three years from different people in different laboratories and finally sequenced on different sequencing runs and different sequencer.
Blinding	N/A

Did the study involve field work? ☒ Yes ☐ No

Field work, collection and transport

Field conditions	The samples were collected from the different fields worldwide following the grapevine-vegetative cycle and consistently sampled within 2 weeks from grape-harvest
Location	Samples were taken from vineyards in California, Spain, Italy, France, Germany, Denmark, Portugal, Australia, Oregon, Texas, Argentina, Chile, Croatia and Hungary
Access & import/export	Samples were collected upon agreement with the owner of the land and exported in compliance of actual laws
Disturbance	no disturbance were caused by this study

Reporting for specific materials, systems and methods

We require information from authors about some types of materials, experimental systems and methods used in many studies. Here, indicate whether each material, system or method listed is relevant to your study. If you are not sure if a list item applies to your research, read the appropriate section before selecting a response.

Materials & experimental systems

n/a	Involved in the study
☒	☐ Antibodies
☒	☐ Eukaryotic cell lines
☒	☐ Palaeontology and archaeology
☒	☐ Animals and other organisms
☒	☐ Human research participants
☒	☐ Clinical data
☒	☐ Dual use research of concern

Methods

n/a	Involved in the study
☒	☐ ChIP-seq
☒	☐ Flow cytometry
☒	☐ MRI-based neuroimaging
